# The awakening of the CDK10/Cyclin M protein kinase

**DOI:** 10.18632/oncotarget.15024

**Published:** 2017-02-02

**Authors:** Vincent J. Guen, Carly Gamble, Jacqueline A. Lees, Pierre Colas

**Affiliations:** ^1^ David H. Koch Institute for Integrative Cancer Research, Massachusetts Institute of Technology, Cambridge, United States of America; ^2^ P2I2 Group, Protein Phosphorylation and Human Disease Laboratory, Station Biologique de Roscoff, Centre National de la Recherche Scientifique, Université Pierre et Marie Curie, Roscoff, France

**Keywords:** CDK10, Cyclin M, ETS2, ciliogenesis, STAR syndrome

## Abstract

Cyclin-dependent kinases (CDKs) play important roles in the control of fundamental cellular processes. Some of the most characterized CDKs are considered to be pertinent therapeutic targets for cancers and other diseases, and first clinical successes have recently been obtained with CDK inhibitors. Although discovered in the pre-genomic era, CDK10 attracted little attention until it was identified as a major determinant of resistance to endocrine therapy for breast cancer. In some studies, CDK10 has been shown to promote cell proliferation whereas other studies have revealed a tumor suppressor function. The recent discovery of Cyclin M as a CDK10 activating partner has allowed the unveiling of a protein kinase activity against the ETS2 oncoprotein, whose degradation is activated by CDK10/Cyclin M-mediated phosphorylation. CDK10/Cyclin M has also been shown to repress ciliogenesis and to maintain actin network architecture, through the phoshorylation of the PKN2 protein kinase and the control of RhoA stability. These findings shed light on the molecular mechanisms underlying STAR syndrome, a severe human developmental genetic disorder caused by mutations in the Cyclin M coding gene. They also pave the way to a better understanding of the role of CDK10/Cyclin M in cancer.

## INTRODUCTION

Cyclin-dependent kinases (CDKs) form a family of 20 serine/threonine protein kinases that play pivotal roles in the regulation of a variety of fundamental cellular processes such as cell division, differentiation, migration and death through the control of transcription, splicing, and protein interactions, localization, stability, etc. [1, 2]. Some CDKs (and especially those that promote cell division) are considered to be potentially interesting therapeutic targets to pursue for pathologies involving uncontrolled cell proliferation including cancer. A cornucopia of small-molecule inhibitors have been discovered and a subset evaluated in patients [3, 4]. Although most tested inhibitors have produced disappointing results, recent successful clinical trials of selective CDK4/6 inhibitors against hormone-dependent breast cancer confirm that targeting CDKs can be a viable therapeutic strategy [5].

CDK10 was discovered as early as 1994 by sequence homology screening for CDK-related genes [6, 7]. It displays the central hallmarks of CDKs, bearing more than 40% sequence identity with CDK1 and other members of the family. Its closest paralog is CDK11, which promotes tumor cell proliferation [8, 9]. Although discovered in the pre-genomic era, and at a time when the study of CDKs yielded major breakthroughs in the understanding of cell cycle regulation, CDK10 received little interest until it was identified as a major determinant of resistance to endocrine therapy for breast cancer [10]. However, for the past twenty years and until recently, the elucidation of the functions of CDK10 was hampered by the lack of any identified cognate cyclin partner, in absence of which no kinase activity can be revealed.

## CDK10 PROMOTES CELL PROLIFERATION

**Figure 1:**
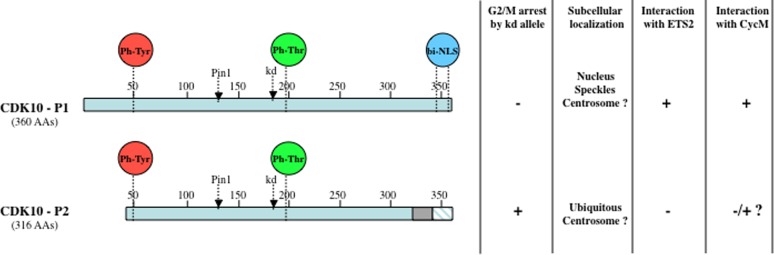
Schematic diagram and properties of two CDK10 splice isoforms Both CDK10 isoforms contain the conserved amino acid residues that undergo regulatory (de)phosphorylation events controlling the (de)activation of CDK proteins. Proteomic studies revealed that CDK10 was phosphorylated on tyrosine 50 (equivalent of tyrosine 15 on CDK1) and tyrosine 54 [89], as well as on threonine 196, located in the activation loop [15, 16, 99–101]. The positions of the Threonine 133 residue required for Pin1 binding [27] and of the Aspartate 181 residue that is mutated to create kinase dead alleles are indicated. A functional bipartite nuclear localization sequence (NLS) is also present at the carboxy-terminus of the longest isoform only [88]. The shorter isoform (CDK10-P2) consists of a protein that, compared to the longest isoform (CDK10-P1), presents an amino-terminal truncation of 29 amino acids, a carboxy-terminal internal deletion of 17 amino acids (grey box) and a carboxy-terminus that differs on the last 15 amino acids (blue hatched box) [7]. Both isoforms originate from alternative splicing of the CDK10 gene, which comprises 14 exons and undergoes complex splicing events involving cryptic splice sites, exon skipping, exon scrambling and insertion of intronic sequences [76]. Most splice variants are suspected to correspond to nonfunctional messengers.

Early work established that CDK10 promotes cell proliferation. A kinase dead (kd) mutant of the shorter isoform of CDK10 (Figure 1) was constructed by mutating the crucial aspartate amino acid located within the conserved ATP binding site of CDKs that is required for the coordination of Mg2+-ATP and thus kinase activity [11, 12]. Such dominant-negative mutants, which have been used to probe the functions of CDK1, 2 and 3 [13], are thought to sequester interaction partners (especially activating cyclins) in enzymatically inactive complexes [14]. The stable expression of CDK10kd in osteosarcoma (U2OS, Saos-2) and glioblastoma (T98G) derived cell lines decreased tumor cell growth in colony formation assays. A decrease in proliferation, albeit smaller, also resulted from expression of a CDK10 antisense cDNA, but not a control, sense construct [12]. The transient expression of CDK10kd in U2OS cells caused a significant increase in the fraction of cells in the G2/M phase, which was comparable to the increase resulting from expression of a CDK1kd mutant [12]. This finding strongly suggested that CDK10 exerts a positive control on cell division, acting during the G2 or M phase of the cell cycle. In line with this hypothesis, subsequent studies showed that phosphorylation of CDK10 on threonine 196, a post-translational modification that serves as an essential activation mechanism for CDKs, was present at similar levels in S-phase and nocodazole arrested (pro-metaphase) cells, but increased dramatically 30 min after release from nocodazole (which corresponds to late M-phase) [15, 16]. These observations suggest that CDK10 activity peaks in late mitosis and thus exerts an important function at this phase [15].

Somewhat surprisingly, expression of a kd allele of murine CDK10 that is nearly identical to the longest human CDK10 isoform, did not inhibit tumor cell growth in a colony formation assay and did not affect the cell cycle profile of U2OS, NIH3T3 or L929 tumor cell lines [17]. Unfortunately, whether the lack of phenotypic effects reflects definitive differences in the role of longer versus shorter CDK10kd isoforms has not been addressed.

More recent studies have used transient RNA interference to silence CDK10 expression in various human cell lines, yielding rapid inhibition of proliferation in Hela cells [18], accumulation in G2 phase in HCT116 colon carcinoma cells [19] or G2/M phase in immortalized retinal pigment epithelial cells [20] and a modest decrease in cell survival, accompanied by a slight activation of caspases 3 and 7, in MCF7 cells (derived from a ERα-positive breast tumor) [10]. Importantly, the question of which CDK10 isoforms were expressed (and thus silenced by the siRNAs) in these cell lines remains open.

## CDK10 REGULATES TRANSCRIPTION AND DEVELOPMENT

A yeast two-hybrid screening conducted against CDK10 identified the ETS2 transcription factor as an interacting protein [21]. This interaction involves the so-called Pointed (PNT) domain of ETS2, located at the amino-terminal region of the protein. The CDK10-ETS2 interaction was confirmed in mammalian cells by co-immunoprecipitation of the ectopically expressed proteins [21] and subsequently the endogenous proteins [10]. Since the PNT domain of ETS2 functions as a transcriptional activation domain, CDK10 was examined for its ability to modulate the transcriptional activity of a chimeric transcription factor, consisting of a fusion between the ETS2 PNT domain and the GAL4 DNA binding domain. Co-expression of CDK10 and this chimera in human cells caused a significant inhibition of the transcription of a reporter gene. Notably, the same degree of transcriptional inhibition was obtained by co-expressing CDK10kd, suggesting that suppression of ETS2's transcriptional activity does not depend on the kinase activity of CDK10 [21].

In an effort to identify determinants of resistance to endocrine therapy for breast cancer, a siRNA screen against the whole human kinome revealed CDK10 as a target whose silencing allowed cultured MCF7 breast cancer cells to grow efficiently in the presence of tamoxifen. The observed drug resistance was caused by upregulation of c-RAF, one of the target genes of ETS2, and consequent activation of the MAP kinase pathway. Chromatin immunoprecipitation experiments established that CDK10 and ETS2 bind to a putative ETS2-binding site in the c-RAF gene promoter, and that CDK10 silencing increases binding of ETS2 to this site [10]. The upregulation of c-RAF in response to inhibition of CDK10 expression has also been observed in biliary tract cancer cell lines [22] and in zebrafish embryos [23].

The developmental functions of CDK10 were explored using zebrafish as a vertebrate model. Inhibiting CDK10 expression by morpholinos hindered the development of the central nervous system by triggering apoptosis in the brain and dorsal neurons. The control exerted by CDK10 on neural progenitor cells was shown to be mediated by the Raf-MEK-ERK1/2 pathway [23]. The developmental functions of CDK10 were also investigated in the lepidopteran Helicoverpa armigera, which poses a severe threat to crop production worldwide. Inhibiting CDK10 expression by RNA interference caused major abnormalities in larvae and delays in pupation and adult transition time. It also downregulated the expression of key target genes of the 20-hydroxyecdysone (20E) steroid hormone, which initiates molting and metamorphosis in insects. 20E was shown to induce CDK10 phosphorylation (on unidentified residues), which itself increased the binding of CDK10 to Hsc70 and Hsp90. These two chaperones are required by the ecdysone receptor (EcR) to bind to its DNA response elements. It was shown that CDK10 participates in the transcription complex that includes Hsc70, Hsp90 and EcR, and that it promotes its interaction with DNA [24]. Thus, CDK10 is a direct participant in at least two distinct transcriptional complexes that play crucial roles in development.

## THE AWAKENING KISS OF CYCLIN M

A yeast two-hybrid screening against CDK10 has revealed an interaction phenotype with the FAM58A gene product, a member of the cyclin protein family [25]. Following the nomenclature proposed in the aftermath of the human genome sequencing [26], this protein was named Cyclin M (CycM). The interaction between CDK10 and Cyclin M was confirmed by co-immunoprecipitation of ectopically overexpressed and subsequently endogenous proteins from human cells. Both proteins showed increased expression levels when their partner was exogenously coexpressed and, remarkably, siRNA-mediated silencing of Cyclin M caused a drastic reduction of CDK10 expression level [25]. These observations suggest that CDK10 and Cyclin M enhance each other's stability. CDK10 has been shown to be subject to ubiquitin-mediated degradation [27], and Cyclin M interaction may protect CDK10 from such degradation. These findings also suggest that Cyclin M is the only conventional cyclin partner of CDK10 in MCF7 cells, in which an interaction between CDK10 and the unconventional Cyclin G2 has recently been reported [28].

Analysis of recombinant purified CDK10/CycM complex produced in insect cells established that CDK10 is a bona fide cyclin-dependent kinase [25]. Specifically, purified CDK10/CycM but not CDK10 alone was able to phosphorylate the canonical CDK substrate Histone H1, and also ETS2, in vitro. The possible role of CDK10/CycM in regulating ETS2 by phosphorylation was explored. First, silencing of either CDK10 or Cyclin M caused a strong increase in ETS2 protein expression levels, without affecting mRNA levels. Accordingly, ectopic expression of CDK10 alone, or together with Cyclin M, decreased the expression level of ectopically expressed ETS2, in starck contrast to the lack of effect of the kinase dead allele of CDK10. Treating cells with a proteasome inhibitor largely rescued ETS2 expression levels, indicating that the CDK10/CycM kinase positively regulates ETS2 degradation via the proteasome. A mass spectrometry analysis of recombinant ETS2 protein phosphorylated in vitro by CDK10/CycM identified multiple phosphorylated sites, including two neighboring serines within a phosphopeptidic motif that is reminiscent of a ubiquitin ligase phosphodegron [29]. An ETS2 protein bearing alanine substitutions of these two phospho-serines (ETS2SASA) was generated and its expression levels compared to ETS2wt. In the absence of ectopic CDK10/CycM, ETS2wt and ETS2SASA were maintained at comparable levels, but overexpression of CDK10/CycM resulted in targeted downregulation of ETS2wt but not ETS2SASA [25]. A subsequent study showed that CDK10-mediated phosphorylation of these two ETS2 residues marks the protein for degradation by the COP1/DET1 ubiquitin ligase complex [30]. Moreover, a p53 mutated protein was found to protect ETS2 from this degradation by competing with DET1 for binding to the region of ETS2 that contains these two regulatory CDK10 phosphorylation sites [30]. Thus, CDK10/CycM can promote ETS2 degradation through formation of a COP1/DET1 phosphodegron. Notably, other ubiquitin ligases, including APC-Cdh1 [31] and CRL4 [32] have also been implicated in ETS2 degradation, independently from CDK10/CycM phosphorylation.

Thus, it appears that CDK10/CycM can restrain ETS2 activity through two distinct mechanisms: activation of ETS2 degradation that is mediated by COP1/DET1 in a kinase-dependent manner, and direct participation in, and repression of, the ETS2 transcriptional complex in a kinase-independent manner.

## CDK10/CYCM MAINTAINS ACTIN NETWORK ARCHITECTURE AND REPRESSES CILIOGENESIS

Primary cilia are microtubule-based organelles that protrude from the surface of most mammalian cell types to sense environmental cues that control development and maintain adult tissue homeostasis [33]. CDK10 and Cyclin M were found to localize to basal bodies (the centrosomal structures that are responsible for the formation and the elongation of primary cilia [34]), prompting the investigation of the potential role of CDK10/CycM in regulating ciliogenesis [20]. RNAi-mediated silencing experiments were performed in human telomerase reverse transcriptase-immortalized retinal pigmented epithelial (hTERT RPE-1) cells, which readily grow primary cilia in response to serum withdrawal [35]. These experiments established that CDK10 and Cyclin M repress primary cilium assembly and elongation in serum-starved cells in a cell-cycle-independent manner, and that both proteins enable cilia disassembly and cell cycle re-rentry in response to mitogenic signaling. CDK10 or Cyclin M silencing did not cause major alterations in the structure of the basal bodies but instead resulted in loss of actin stress fibers, which are known to inhibit ciliogenesis [36, 37]. To understand the molecular mechanisms by which CDK10/CycM represses ciliogenesis and maintains actin network architecture, an unbiased screen was performed to identify in vitro phosphorylation substrates of the kinase, using a array of almost 10,000 recombinant proteins. All five positive hits obtained were known regulators of actin dynamics [20]. The strongest hit, protein kinase C-like 2 (PKN2, also known as protein kinase C-related kinase 2 or PRK2), was further pursued, owing to its known interaction with RhoA and its positive role in various RhoA-regulated processes, including stress fiber formation [38]. This analysis revealed PKN2 as a novel repressor of ciliogenesis via RhoA regulation. Specifically, depletion of CDK10/CycM, depletion of PKN2, or expression of a PKN2 mutant that cannot be phosphorylated by CDK10/CycM resulted in the degradation of the RhoA protein. Importantly, ectopic expression of RhoA was shown to override the effect of CDK10/CycM silencing on ciliogenesis, thereby establishing that CDK10/CycM exerts its ciliogenesis function through the PKN2-RhoA pathway [20].

## CDK10/CYCM AND STAR SYNDROME

Cyclin M had never attracted any kind of attention until loss-of-function mutations on its gene FAM58A were shown to cause severe, multiple malformations in young girls [39]. This so-called STAR syndrome includes syndactyly (fusion of two or more digits), telecanthus (abnormally large distance between the eyes), anogenital and renal malformations, general growth retardation. Additional severe skeletal, ocular, pulmonar and cardiological defects can be observed [39–43]. So far, studies on a total of 11 patients have identified various heterozygous molecular lesions affecting the FAM58A gene, which is located on the X chromosome. These lesions include deletions affecting single or multiple exons, point mutations affecting splice donor or acceptor sites, point mutations introducing a frameshift mutation or a stop codon [39, 41, 43], or a large genomic loss at Xq28 affecting multiple genes including FAM58A [42]. At this point, it is unclear whether some of these mutations give rise to truncated proteins, or whether the mutant transcripts are eliminated by a non-sense mediated decay (NMD) mechanism [44]. Regardless, two C-terminal truncated forms of Cyclin M corresponding to the hypothetical translation products of two mutated genes identified in STAR syndrome patients were unable to bind to CDK10 in two-hybrid interaction assays [25]. This suggests that in STAR patients, the CDK10/CycM protein kinase activity is compromised at least in some tissues and/or developmental stages, depending on which X chromosome is inactivated. To verify this hypothesis, ETS2 expression levels were examined in a lymphoblastoid cell line obtained from a STAR syndrome patient exhibiting incomplete skewing of X chromosome inactivation [39]. Cyclin M protein and mRNA levels were found to be decreased (as compared to those detected in a control cell line), suggesting that the FAM58A mutation carried by this patient causes NMD. The mRNA level of ETS2 was identical to that of the control cell line, but in accordance with the effects of Cyclin M knockdown, the protein level of ETS2 was increased. This increase was attributed to the lowered level of Cyclin M, since ectopic expression of Cyclin M in the patient-derived cells caused a decrease in ETS2 protein levels [25].

These findings shed light on the molecular mechanisms underlying STAR syndrome. Ets2 transgenic mice showing a less than two-fold overexpression of Ets2 exhibit cranial abnormalities, one of the features affecting STAR patients [45]. Because ETS2 dosage can repress or promote tumor growth, elevated levels of ETS2 in STAR patients might protect them from the occurrence of certain types of cancers, and might increase their chances of developing others. Interestingly, of the eleven STAR patients identified to date, two have developed a nephroblastoma [43, 46]. The recent study showing that CDK10/CycM represses ciliogenesis brings another important contribution to the understanding of the etiology of STAR syndrome [20]. Ciliary defects offer a plausible explanation of various developmental disorders in STAR patients, such as the renal, retinal, anogenital and digital anomalies, which are frequently observed in ciliopathies. Consistent with this hypothesis, analysis of a renal biopsy taken from a STAR patient [46] revealed dilated tubules and abnormal, elongated cilia as compared to a non-STAR control [20].

Hence, the combination of enhanced ETS2 levels and ciliary defects resulting from compromised CDK10/CycM activity may explain most, if not all, of the clinical features of STAR syndrome. As reasonable as this hypothesis may seem, CDK and cyclin gene knockout studies in mice call for caution. Some proteins within these two families display such functional redundancies that a single gene knockout can often trigger compensatory mechanisms, which result in a lack of conspicuous phenotypes [47]. Hence, the absence of Cyclin M could be partially or fully compensated by another cyclin, and the malformations caused by the loss-of-function of Cyclin M could (also) result from the deregulation of other Cyclin M interacting partners, such as the transcriptional repressor SALL1 [39].

## CDK10/CYCM AND CANCER

### Expression studies

A number of transcriptomic and proteomic studies report upregulation of CDK10 in cancer cells or in cells exhibiting exacerbated division, and/or downregulation of CDK10 in differentiated cells [48–56] (Table 1). For example, CDK10 was found upregulated in tumor prostate specimens [49] and seminomas [50]. Conversely, CDK10 was found downregulated in retinoic acid-treated retinoblastoma cells [53] and in butyrate-treated colon carcinoma cells [57], where both treatments trigger cell cycle arrest. CDK10 transcripts were found to be downregulated in a multiple myeloma cell line treated with a histone deacetylase inhibitor that is currently being evaluated in clinical trials [48]. Another interesting study suggested that CDK10 may participate in the resistance of tumor cells to p53-mediated apoptosis [55]. The concordance of these observations is challenged by a nearly equal number of studies that reveal downregulation of CDK10 messengers or protein in cancer cells and/or upregulation in non-dividing cells [22, 58–65] (Table 2). For example, CDK10 mRNA and/or protein levels were found downregulated in biliary tract carcinomas [22], hepatocellular carcinomas [58], low-grade and, to a lesser extent, high-grade glial tumors [59] and breast cancer tissues compared to adjacent noncancerous tissues [64]. In the latter study, the decreased CDK10 protein levels were associated with lymph node metastasis and unfavorable overall survival.

**Table 1:** Expression studies reporting a positive correlation between CDK10 expression and cell division and/or tumoral state

Comparative study	Scope	Differential expression	Ref
Multiple myeloma cell line treated with histone deacetylase inhbitor *vs* untreated	38,500 gene microarray	Downregulated (2x)	[48]
Malignant *vs* Benign prostate specimens	7068 gene microarray	Upregulated (13.4 x) in 9 of 11 tumors *vs* all 4 benign samples	[49]
Seminomas *vs* Normal testicular tissues	Nuclear matrix proteins	Upregulated – confirmed by Western blot experiments	[50]
Mantle cell lymphoma (MCL) with mutated or deleted ATM gene *vs* MCL with wt ATM	12,196 cDNA microarray	Upregulated (1.27x)	[51]
Stenotic saphenous aorto-coronary grafts *vs* Ungrafted saphenous vein segments	91 cDNA array	Upregulated (> 2 x) in 3/5 tested veins	[52]
RA-induced differentiated retinoblastoma cells *vs* Untreated retinoblastoma cells	6,800 gene microarray	Downregulated (10.8 x)	[53]
Lung adenocarcinoma *vs* Non-neoplastic pulmonary tissue	44,363 gene microarray	Upregulated (1.5 x)	[54]
p53-mediated apoptosis-resistant *vs* Apoptosis-sensitive bladder carcinoma cell lines	5730 gene microarray	Upregulated (2x) – confirmed by RT-PCR experiments	[55]
Follicular lymphomas *vs* Normal germinal center B cells	588 cDNA array	Upregulated (1.3 x) – confirmed by real time quantitative RT-PCR	[56]

**Table 2:** Expression studies reporting a negative correlation between CDK10 expression andcell division and/or tumoral state

Comparative study	Scope	Differential expression	Ref
Biliary tract tumor samples *vs* Normal tissues	qRT-PCR on 47 tumor samples Wb on 18 tumor samples	mRNA downregulated in 77% of samples Protein downregulated in 83% of samples	[22]
Hepatocellular carcinomas *vs* Adjacent non-tumoral liver tissues	qRT-PCR on 127 specimen Tissue immunostaining	mRNA downregulated Protein downregulated in 70% of samples	[58]
Gliomas *vs* Normal glial tissue	114 cell cycle gene macroarray	Downregulated (5x and 1.9x) in low and high grade tumors	[59]
Peritoneal-metastatic cell line variants *vs* Parental low-metastatic cell lines	2000 gene microarray	Downregulated (8 x)	[60]
Senescent *vs* Young primary fibroblasts Quiescent *vs* Young primary fibroblasts	Genes on the long arm of chromosome 16 terminal region	Upregulated (8 x) Upregulated (18 x)	[61]
3 endometrial cancer cell lines infected with PTEN expressing virus *vs* Empty virus	4009 cDNA array	Upregulated (2.2 to 8.7 x) – confirmed by RT-PCR	[62]
Human kidney cells with activated PAR2 *vs* Non-activated	19,000 gene microarray	Downregulated (up to 2x) in two PAR2-activating conditions	[63]
Breast cancer tissue *vs* Adjacent nontumoral tissue	Wb on 20 paired tissues IHC on 128 tumor tissues	Decreased levels in 65/128 tumor tissues	[64]
Primary nasopharyngeal carcinomas *vs* Chronic nasopharyngitis samples	Semi qRT-PCR on 40 NPC and 5 nasopharyngitis samples	mRNA downregulated in 57% of tumor samples	[65]

### CDK10 can act as a tumor suppressor

In apparent contradiction with its documented positive role in cell cycle regulation, CDK10 was found to act as a tumor suppressor in a number of tumor cells. Stable overexpression of CDK10 in a gallbladder or a cholangiocarcinoma cell line markedly inhibited cell proliferation and migration, and increased the sensitivity of both cell lines to the chemotherapeutic agents 5-FU, EADM, CDDP, HCPT. Moreover, CDK10 silencing produced opposite effects on proliferation, migration and drug response [22]. Transient overexpression of CDK10 in human hepatocellular carcinoma cell lines also caused an inhibition of cell proliferation, cell migration and anchorage-independent growth, and it increased sensitivity to cisplatin and epidoxorubicin [58]. In both studies, CDK10 overexpression caused an increase and a decrease in the G1 and S-phase cell populations, respectively [22, 58]. CDK10 ectopic expression in a nasopharyngeal cell line strongly inhibited growth and invasion [65].

### CDK10 and hormone-dependent breast cancer

The clinical significance of the involvement of CDK10 in the response of MCF7 breast cancer cells to tamoxifen was investigated by measuring CDK10 expression levels in tumors from breast cancer patients subjected to an endocrine therapy [10]. A data mining effort was first conducted on a prior study that aimed at defining clinically distinct subtypes in estrogen receptor-positive breast carcinomas through gene expression profiling of 87 tumors from patients treated with adjuvant tamoxifen [66]. A statistically significant association was found between low CDK10 expression level and shorter time to distant relapse of disease. To validate this finding, the authors performed a de novo analysis on a second set of 38 tumors, in which CDK10 expression levels were measured by quantitative PCR. This confirmed the statistically significant association between low CDK10 and shorter time to disease progression, and extended it to poor patient survival. In both cohorts, an association between low CDK10 expression and well-established prognostic factors (age, tumor size, grade, etc.) or the expression of canonical biomarkers (HER2, MIB1, p53, ERα, PR) was excluded. Finally, to account for the decreased expression of CDK10 in some patients, the methylation status of the CpG island in the CDK10 gene promoter was examined on the second tumor set. Methylation was detected in 7 of the 38 cases, and methylation was strongly associated with low CDK10 expression, shorter time to disease progression, and shorter overall survival [10]. Methylation was also detected in primary nasopharyngeal carcinomas and was found to correlate with downregulation of CDK10 expression [65]. However, another study of 96 breast carcinoma patients failed to detect methylation of the CDK10 gene promoter [67]. Thus, doubt remains regarding the role of promoter methylation in the reduced expression of CDK10 in some hormone-dependent breast cancer patients.

Post-translational control is likely to be involved in the regulation of CDK10 levels. The peptidyl-prolyl isomerase Pin1, which facilitates the progression of tamoxifen resitance [68], was shown to interact with CDK10 and promote its ubiquitin-mediated degradation [27]. At the post-transcriptional level, the role of miRNA-mediated regulation is also worth exploring. CDK10 transcripts were detected in miR-210-enriched RISC complexes in HEK-293 cells, and were found to be downregulated in HUVEC cells exposed to hypoxia, which strongly induces miR-210 expression [69]. Interestingly, miR-210 is overexpressed in lymph node- negative, estrogen receptor-positive breast cancers, and a correlation is observed between its expression level and the aggressiveness of the tumors [70]. Moreover, miR-210 expression appears to represent a good prognostic marker of the patients under tamoxifen treatment [71]. Although miR-210 overexpression or repression was not found to significantly modify CDK10 expression in two breast cancer cell lines [71], miR-210 may play an important role in controlling CDK10 expression in vivo. Regardless of the underlying cause(s) of its reduced expression, CDK10 appears to be a promising biomarker to predict the response of ER-positive breast cancer patients to endocrine therapy and, for some of them, to prescribe alternative treatments that may be more effective.

### CDK10: a biomarker for gastro-intestinal cancers?

A number of studies suggest that CDK10 expression level could represent a relevant biomarker to characterize some gastro-intestinal tumors. The analysis of a collection of human biliary tract tumors revealed that in 70% of the samples, increased c-RAF levels and concomitant decreased CDK10 mRNA levels associated with worse TNM staging and increased lymph node invasions [22]. Immunohistochemical studies of hepatocellular carcinomas showed that decreased CDK10 protein expression levels were significantly correlated with tumor size, alpha-fetoprotein levels, and tumor stage [58]. The CDK10 gene was mapped to chromosome 16 at location q24, a region that shows loss of heterozygosity (LOH) in a number of cancers including hepatocellular carcinomas [72].

### CDK10 and cutaneous melanomas

A recent meta-analysis of genome-wide association studies (GWAS) on cutaneous melanomas (compiling about 200 studies, >80,000 cases, >1100 polymorphisms on <300 genes) identified CDK10 amongst the 10 loci showing a single-nucleotide polymorphism (SNP) found in significant association with this cancer [73], in accordance with previous studies [74, 75]. This polymorphism lies in an intron and may affect the highly complex splicing of CDK10 [76], as has been shown for another neighboring intronic SNP [77].

## SUSPECTED ADDITIONAL FUNCTIONS OF CDK10/CYCM IN SPLICING AND TRANSCRIPTIONAL REGULATION

Cyclin M exhibits strong sequence homology to the so-called transcriptional cyclins, and especially to the L-type cyclins that associate with CDK11, 12 and 13 to regulate transcription and splicing [78]. In two independent studies, CDK10 was found among the 150 proteins forming the ribonucleoprotein (RNP) core of the human spliceosomal C complex, which catalyzes the second step of splicing that consists of intron excision and exon ligation [79, 80]. CDK10 was not found in more precocious spliceosomal complexes such as the human activated B complex [80] or the human and fly spliceosomal B complexes, except when a short intron-containing pre-mRNA was used to purify Drosophila B complexes [79, 81]. In all the above-mentioned studies, Cyclin M was only weakly detected in human activated B complexes [80] and neither CDK11 nor Cyclins L were detected. However, CDK11 was detected in human purified prespliceosomal A complexes [82] and in purified B complexes [83]. These proteomics studies suggest that CDK10 and 11, which are more homologous to one another than to any other member of the CDK family, may play distinct, sequential roles in gene splicing.

The STAR phenotype shares many common features with that of Townes-Brocks syndrome, caused by mutations in the SALL1 gene [84]. SALL1 belongs to a family of multi-zinc finger proteins that control organogenesis. An interaction between Cyclin M and SALL1 was detected in human cells coexpressing both proteins [39]. Although the functional relevance of this interaction remains to be established, it is tempting to suggest an analogy with the SALL4-Cyclin D1 complex and to speculate that Cyclin M may potentiate SALL1 transcriptional repressor activity [85]. This activity is negatively controlled by Protein kinase C, which phosphorylates a crucial residue in the repression domain [86]. Phosphorylation by CDK10/CycM could also modulate SALL1 repressor activity.

## FORESEEABLE CHALLENGES FOR FUTURE STUDIES

**Figure 2:**
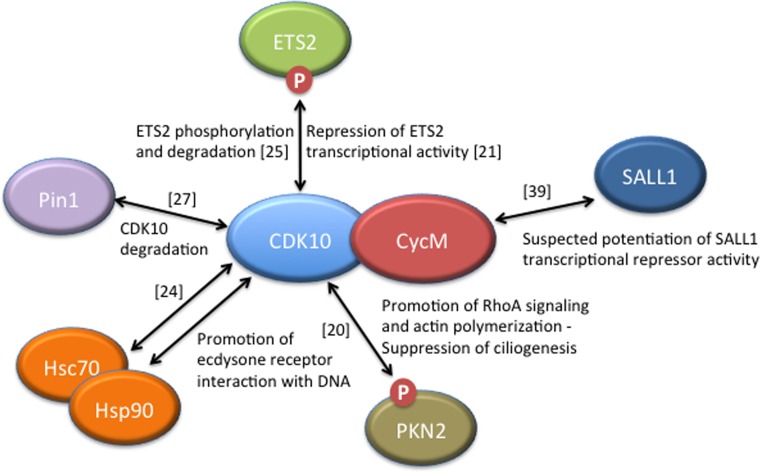
CDK10/CycM protein interactions and associated functions Only those interactions for which functional data have been obtained are included, with the exception of SALL1, for which strong human genetics evidence suggests a biologically-relevant interaction with Cyclin M. Those interacting proteins that have been shown to be phosphorylated by the CDK10/CycM protein kinase are labelled with a ”P”. References reporting the discovery of these interactions are noted.

As exemplified recently [20], the identification of an activating cyclin partner of CDK10 and consequent unveiling of its protein kinase activity will facilitate the elucidation of the biological functions of this still largely mysterious protein and the discovery of additional phosphorylation substrates and interacting partners (Figure 2). However, further studies will face important challenges and difficulties.

First, the different splice isoforms of CDK10 are likely to play different, perhaps opposing roles, as frequently observed for many proteins exerting important functions in cancer [87]. The shorter splice isoform (Figure 1) does not interact with ETS2 and may interact only very weakly with Cyclin M [25]. It does not contain the carboxy-terminal bipartite nuclear localization sequence [88] that addresses the longest isoform to the nucleus [50, 89]. It remains to be determined which of the two CDK10 isoforms localize to the centrosome [20]. FAM58A is also subject to differential splicing, giving rise to shorter Cyclin M isoforms that lose their ability to interact with CDK10 [25]. Quite interestingly, according to a statistical analysis of a transcriptomic study conducted with a microarray of over one million exons, FAM58A was among the 20 genes that underwent the most significant differential splicing when comparing colon cancers with normal colon tissues [90]. Although another analysis conducted on the same study did not retain FAM58A among the most significant splicing events [91], it appears that both CDK10 and FAM58A can give rise to multiple isoforms playing different roles. This could explain some apparent discrepancies between previous studies and will need to be taken into account in future investigations.

Second, in absence of an identified small-molecule inhibitor, a chemical biology approach cannot be undertaken to probe the functions of CDK10/CycM. The in vitro protein kinase activity of this heterodimer [20, 25] should allow the development of a screening assay to identify new inhibitors and, as importantly, should widen the specificity profiling of already identified CDK inhibitors. Although a structural model of CDK10 has been generated by homology [92], rational design of molecules or structure-guided optimization of inhibitors discovered by screening will require a crystal structure of the heterodimer.

Third, the elucidation of CDK10 and Cyclin M functions will hardly benefit from the use of genetically-tractable organisms, which have often contributed to the study of other CDK/cyclin pairs. Only distant ancestor genes can be found in yeast models and no clear CDK10 or FAM58A orthologs seem to exist in Caenorhabditis. Drosophila does have apparent CDK10 and FAM58A orthologs (respectively cdc2rk and koko) but, so far, no conspicuous phenotypes associated with mutations have been reported. However, fly CDK10 and Cyclin M produced an interaction phenotype in large-scale two-hybrid experiments (see Finley lab website, http://www.droidb.org), and fly CDK10 was detected in purified spliceosomal complexes [81], similar to its human ortholog. Thus, Drosophila might provide a useful model to the study of some conserved functions of CDK10 /CycM. Mouse gene knockouts may produce informative phenotypes but the complex combinatorial interactions between CDKs and cyclins and the multiple compensatory mechanisms often observed among both protein families could blur the interpretation of the results [93].

Finally, the highly complex contributions of ETS2 on the one hand, and of primary cilia on the other hand, will obviously complicate the elucidation of the role of CDK10/CycM in tumorigenesis and/or in tumor suppression. ETS2 has been initially described as a proto-oncogene frequently found deregulated in many cancers. Overexpression of ETS2 stimulates cell proliferation, anchorage-independent growth and tumorigenicity in nude mice, and ETS2 knockdown produces opposite effects [94, 95]. The fact that CDK10/CycM promotes ETS2 degradation and inhibits its transcriptional activity would support a tumor suppressor role of the kinase. However, studies on various mouse models (including models of Down's syndrome, in which three copies of ETS2 exist) have revealed that ETS2 can also repress tumor growth [96], and this would support a positive role of the kinase in regulating cell proliferation and tumorigenesis. The sometimes opposite roles of primary cilia in cancer, according to the oncogenic drivers involved, will represent another difficulty [33]. For example, ciliary ablation has been shown to inhibit or to stimulate the progression of basal cell carcinomas [97] and medulloblastomas [98] driven by the Hedgehog pathway activator Smo, or the downstream Hedgehog pathway effector GLI2, respectively.

As with other CDK/cyclin kinases, many years of work will be necessary to achieve a clear understanding of the pleiotropic functions of the CDK10/CycM protein kinase and of its relevance as a potential therapeutic target and/or biomarker in cancers.
